# No evidence that monkeys attribute mental states to animated shapes in the Heider–Simmel videos

**DOI:** 10.1038/s41598-021-82702-6

**Published:** 2021-02-04

**Authors:** Jamie L. Schafroth, Benjamin M. Basile, Alex Martin, Elisabeth A. Murray

**Affiliations:** 1 Section On the Neurobiology of Learning and Memory, Laboratory of Neuropsychology, National Institute of Mental Health, NIH, Building 49, Room 1B80, 49 Convent Drive MSC 4415, Bethesda, MD 20892-4415 USA; 2 Laboratory of Brain and Cognition, National Institute of Mental Health, NIH, Bethesda, MD USA

**Keywords:** Psychology, Animal behaviour

## Abstract

Human Theory of Mind (ToM) is so automatic and pervasive that we spontaneously attribute mental states to animated abstract shapes, as evidenced by the classic Heider–Simmel findings. The extent to which this represents a fundamental characteristic of primate social cognition is debated. Prior research suggests that monkeys spontaneously predict behavior and attribute basic goals to conspecifics, but it remains unclear whether, like humans, they spontaneously ascribe mental states to animated shapes. Here, we address this question by analyzing rhesus monkeys’ viewing patterns of the classic Heider–Simmel animations. We hypothesized that if rhesus monkeys also spontaneously attribute mental states to animated shapes, then, like humans, they would have the longest fixation durations for theory of mind animations, medium duration fixation for goal-directed animations, and shortest fixations for animations with random motion. In contrast, if attributing mental states to animations is specific to humans and perhaps other apes, then we predict no differences in looking time across animation categories. Unlike humans, monkeys did not fixate longer on ToM videos. Critically, monkeys’ viewing patterns did not correlate with humans’ viewing patterns or intentionality ratings from previously published research. The only major difference in viewing patterns between animation categories tracked differences in low-level visual motion. Thus, monkeys do not view the classic Heider–Simmel animations like humans do and we found no evidence that they spontaneously attribute mental states to animated shapes.

## Introduction

Much of human social behavior depends on the ability to represent others’ mental states, such as goals, beliefs, and intentions. This representation is referred to as theory of mind (ToM)^1,2^. For example, we can infer that someone carries an umbrella because she believes it is going to rain. Humans likely develop ToM early in life^3–5^, suggesting that ToM is a fundamental aspect of human cognition.

Human ToM is so automatic and pervasive that we even spontaneously attribute mental states to animated abstract shapes, as evidenced by the classic Heider–Simmel findings^6^. Empirically, the Heider Simmel animations have been shown in humans to elicit different cognitive attributions based on their movement patterns. For example, Abell et al.^7^ showed that if the animated shapes move randomly, then typically developed people give them action-oriented descriptions (e.g., drifting). However, if shapes appear to interact socially, then people spontaneously describe them as having specific goals (e.g., two shapes are chasing each other) or attributing beliefs (e.g., one shape is trying to coax another out from hiding). In the previous study most relevant to the current study, researchers investigated how viewing patterns correlate with the more commonly used verbal reports of intentionality^8^. They used three different animation categories: Theory of Mind (ToM), which showed animated shapes moving in a way that indicated one object acknowledged and reacted to the other object’s mental state; Goal Directed behaviors, which showed animated shapes moving in a purposeful manner but not necessitating the attribution of a mental state; and Random behaviors, which showed animated shapes moving purposelessly. Thus, while both ToM and Goal Directed animations involve purposeful action and interaction, only the former elicited explanations requiring the referencing of mental states (e.g., one shape is fearful and needs to be persuaded out of hiding). ToM animations elicited longer fixations than did Goal Directed animations, which elicited longer fixations than did Random animations, suggesting an increasing depth of processing as subjects attributed goals and mental states^8^. Critically, these systematic differences in viewing correlated with explicit ratings of intentionality, suggesting that viewing patterns of the Heider–Simmel animations can be used as a nonverbal test of mental state attribution to animated shapes. Two major advantages of free-viewing tasks are that they reduce the cognitive and mnemonic load associated with more traditionally used false belief tasks and that they allow the study of preverbal infants, people with verbal impairments, and nonhuman animals^9^.

ToM has been theorized to be a fundamental aspect of cognition for all species that live in sufficiently complex social groups because the evolutionary pressures of social communication likely select for social-specific cognitive processing capabilities^10,11^. Using a comparative approach to study ToM elucidates the degree to which ToM is primarily a product of human evolution. Although preverbal infants and people with verbal impairments demonstrate some ToM abilities on other tasks^3–5^, humans are a fundamentally verbal species. Thus, as this present study uses a nonverbal species, it informs the degree to which language facilitates ToM, complementing previous studies in humans. In addition, it is important to identify and expand ethologically relevant models for neuroscientific investigations of social deficits. Therefore, studying ToM in other primates informs our understanding of the evolution of social cognition and the role of language in ToM.

The bulk of evidence about ToM in nonhuman primates comes from other great ape species. Most great apes generally can attribute knowledge^12–14^, understand and assume that others make inferences based on their own previous experience^15^, and attribute goals and intentions to both conspecifics^16^ and to abstract moving shapes^17^. In addition, there is evidence both for and against the claim that great apes can attribute false beliefs^18–21^. The strengths and weaknesses of this evidence have been discussed elsewhere^22,23^, but a reasonable conclusion is that great apes possess ToM abilities that largely, but not fully, overlap with human ToM abilities^24,25^.

Relatively less evidence exists in monkeys, which are of particular interest for neuroscientific investigations of social processing disorders, and this evidence suggests that they possess some characteristics of ToM but not full human-like ToM^10^. For example, macaque monkeys prefer to steal from individuals who are unaware of their presence over those that are aware^26^. Both macaque and capuchin monkeys also are more tolerant of humans who appear to be unable to give them food than those who appear unwilling^27,28^ and this behavior is specific to animate motion^28^. Monkeys are also able to discriminate between efficient and inefficient actions^29^ and between accidental and intentional cues about the location of food^30,31^. For example, cotton-top tamarins, rhesus macaques, and chimpanzees all chose targeted food locations more often when the human experimenter intentionally interacted with it than when there was an accidental association between the location and experimenter^30^, but see^31^. However, monkeys fail most false belief tests^32,33^, but see^34^. Together, this evidence suggests that monkeys share some characteristics of ToM with humans, such as goal attribution, but also shows distinct differences, such as a lack of false belief attribution.

To our knowledge, only four studies exist about whether monkeys, like humans, attribute mental states to moving animated shapes. The evidence is mixed. Two studies showed that marmoset monkeys looked longer when observing a conspecific or conspecific-like robot unexpectedly behave in violation of an expected goal, but not if the observed agent was replaced with a black box^35,36^. These findings suggest that marmosets can attribute goals to conspecifics, but not to abstract shapes. Two additional studies used an operant conditioning paradigm to show that squirrel monkeys^37^ and Japanese macaques^38^ that had been trained to respond to moving dots with apparent biological motion also transfer that responding to other dot animations with similar motion. This is consistent with the hypothesis that monkeys do attribute goals to inanimate shapes. However, as the authors themselves note, a limitation of operant paradigms is that monkeys may have learned a reward association based on low-level perceptual features rather than on judgements of goal directedness. In other words, the monkeys might not have attributed goals to the animation, but rather learned over the thousands of training trials they experienced that dots associated with certain movement characteristics are rewarded. Thus, these types of studies are ideally supplemented with studies using spontaneous free-viewing responses.

**Figure 1:**
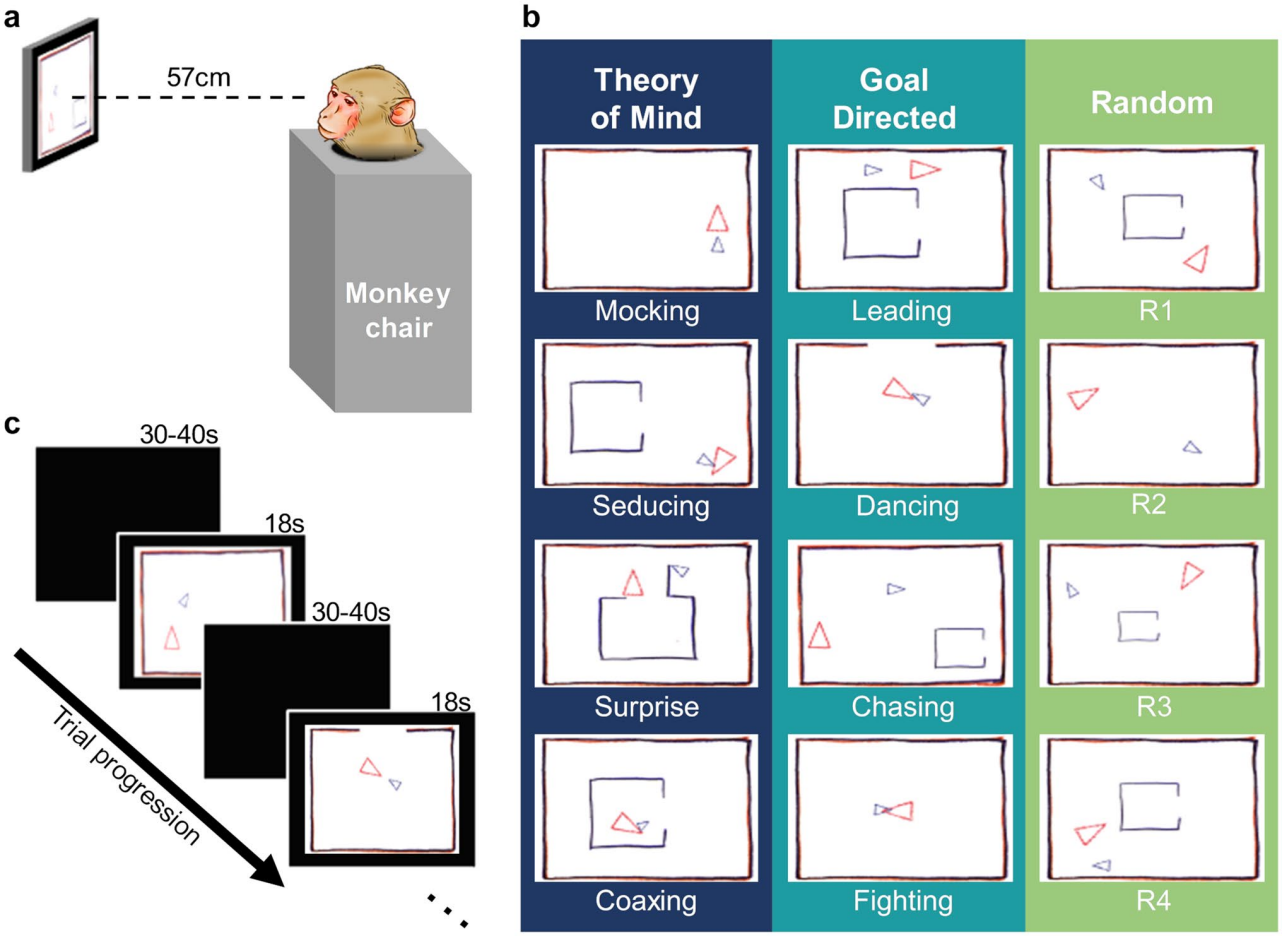
Monkeys freely viewed the Heider–Simmel animations. (**a**) While seated in a primate chair with heads fixed, monkeys viewed 12 animations lasting 18 s each. (**b**) Abell et al.^7^ animations categories: Theory of Mind, Goal Directed, and Random. Each category was composed of four videos. (**c**) Monkeys viewed the animations in a random order with 30–40 s intertrial intervals.

Surprisingly, there are no reports of monkeys having been shown the same Heider–Simmel animations used with humans. Here, we directly compare the viewing patterns of rhesus monkeys (*Macaca mulatta*) to published data on the viewing patterns of normal humans (Fig. 1)^8^. If spontaneously ascribing mental states to abstract animations is a shared characteristic of primate social processing, then we expect rhesus monkeys to view the Heider–Simmel animations in the same way as do humans by showing longer fixations during ToM animations, followed by Goal Directed animations, and then Random animations. This would indicate increased cognitive processing of Goal Directed and ToM videos and would be consistent with attribution of mental states. Thus, this study supplements the existing research in nonhuman primates by providing missing evidence using a classic set of stimuli.

## Results

**Figure 2:**
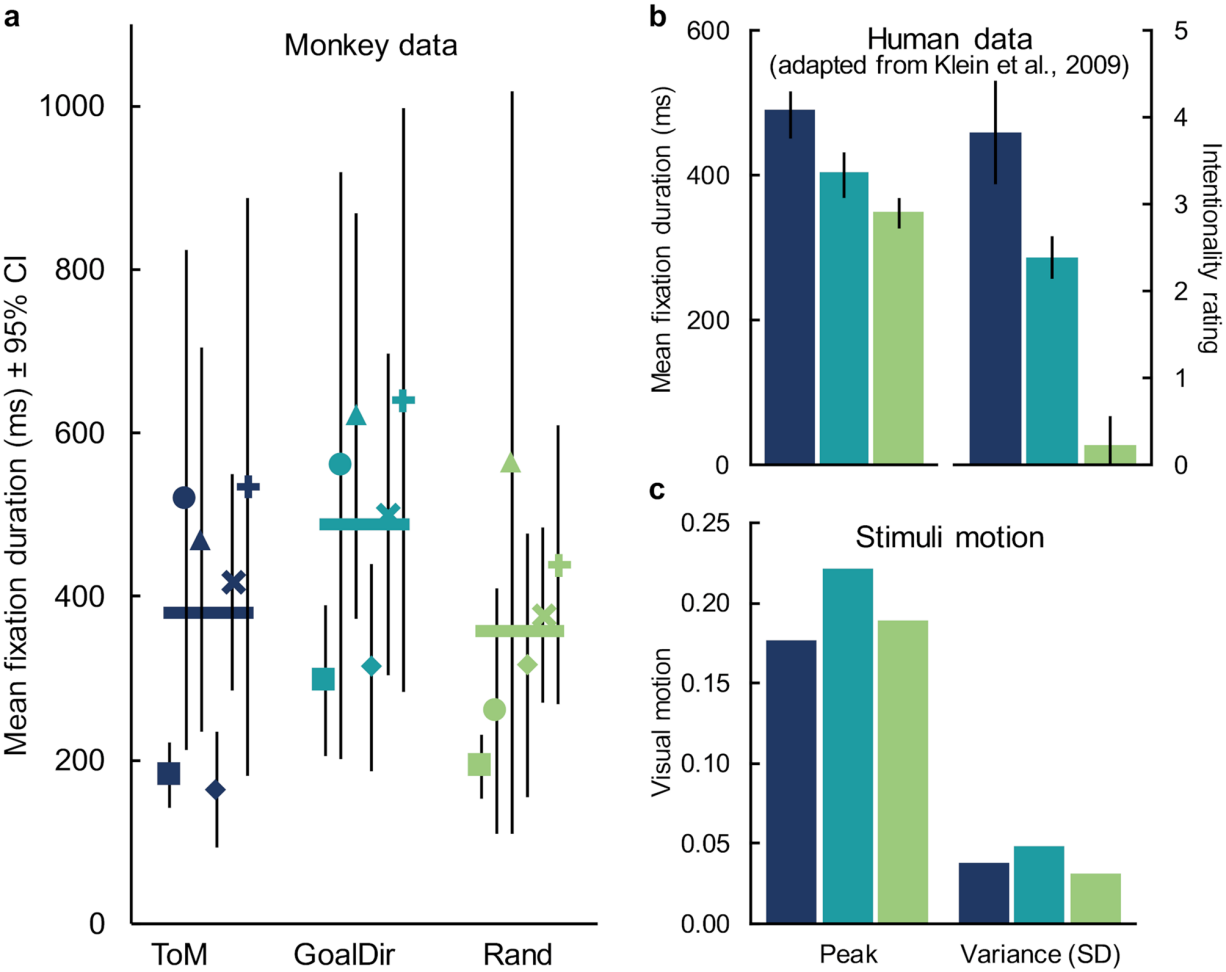
Monkeys did not show longer fixations for ToM videos. (**a**) Monkeys’ fixation durations as a function of animation category. Each thick horizontal line is the group mean fixation duration and each symbol represents an individual monkey’s mean fixation duration with ± 95% CI (ms). Abbreviations: ToM, Theory of Mind; GoalDir, Goal Directed; Rand, Random. (**b**) For comparison, humans’ fixation durations (± 95% CI) and intentionality ratings (± SD) as a function of category. Adapted from Klein et al.^8^. (**c**) Peak visual motion, left, and standard deviation in visual motion, right, of the animations as a function of category. Visual motion was calculated as in^39^.

First, we examined if there were differences in mean fixation duration (ms) as a function of animation category. At the group level, the repeated measures ANOVA revealed a significant difference between categories (Fig. 2a; F_(2,10)_ = 5.06, *p* = 0.030, partial η^2^ = 0.50). However, opposite the pattern in humans (Fig. 2b), monkeys exhibited longer fixation durations when viewing videos in the GoalDir category compared to the ToM category (t_5_ = − 6.32, *p* = 0.001, Bonferroni corrected α = 0.025). Also unlike the pattern in humans, there were no significant differences in fixation duration between the ToM and Rand categories (t_5_ = 0.41, *p* = 0.70, Bonferroni corrected α = 0.025) and the difference between GoalDir and Rand videos was just outside significance when correcting for multiple comparisons (t_5_ = 3.00, *p* = 0.03, Bonferroni corrected α = 0.025).

Because we anticipated that visual motion in the animations might influence fixation duration, we reanalyzed the results using either peak motion or motion variability as a covariate. Importantly, re-running the ANOVA with motion as a co-variate abolished the observed category difference in fixation duration (Fig. 2c; with peak motion: F_(2,10)_ = 0.96, *p* = 0.417; with motion variability: F_(2,10)_ = 0.28, *p* = 0.762).

**Figure 3:**
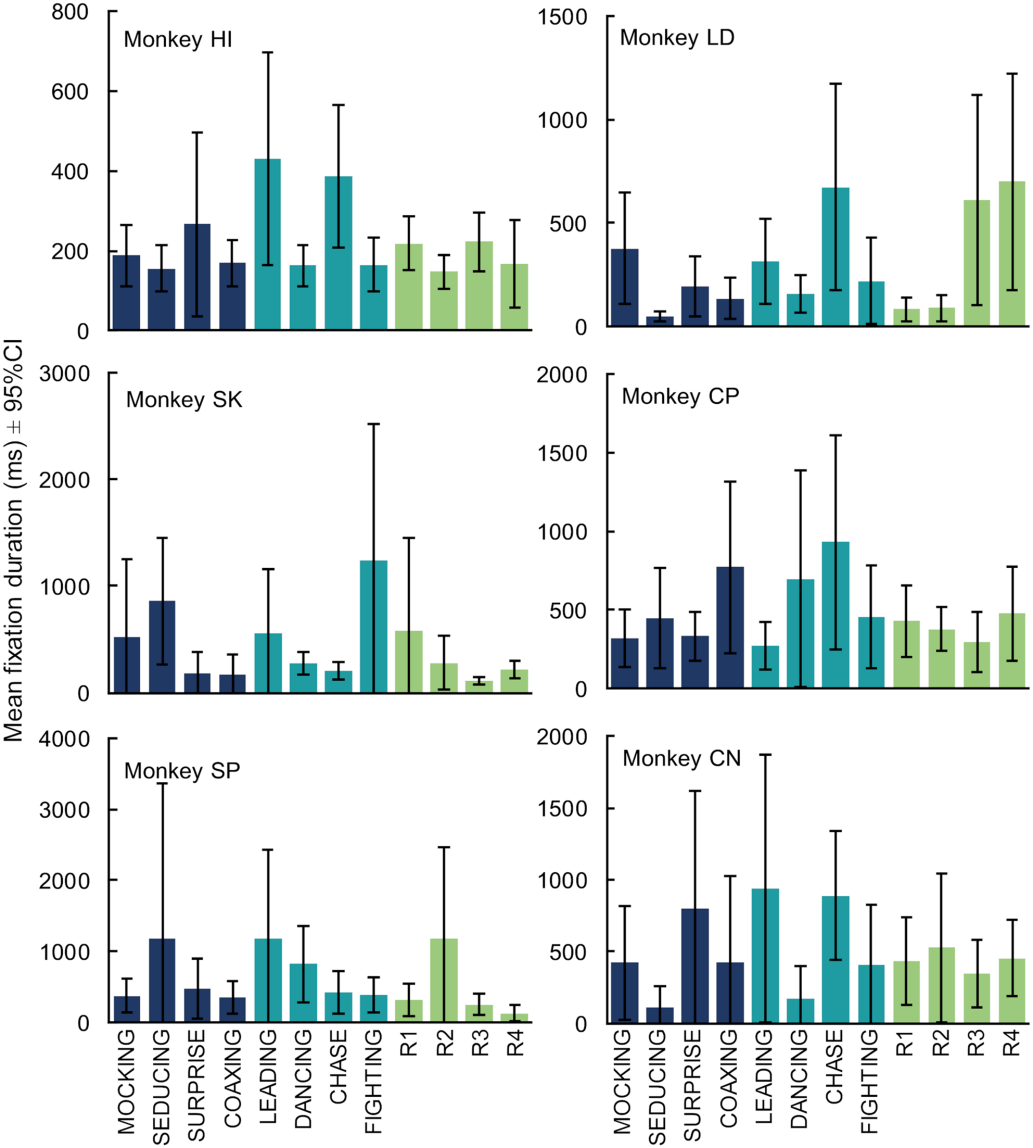
Monkeys viewing preferences were idiosyncratic. Monkeys showed viewing preferences for individual videos, not broad categories. Colors represent video categories as in Figs. 1 and 2. See also Supplemental Fig. 1.

To thoroughly examine the data, we next looked at the performance of each individual monkey (Fig. 3). Five of the six monkeys showed no significant difference between categories (Monkey SK: F_(2, 76)_ = 0.68, *p* = 0.51; Monkey SP: F_(2,94)_ = 0.23, *p* = 0.80; Monkey LD: F_(2, 300)_ = 2.32, *p* = 0.10, Monkey CP: F_(2, 233)_ = 0.71, *p* = 0.50; Monkey CN: F_(2, 116)_ = 0.52, *p* = 0.60). One monkey, Monkey HI, showed a significant difference in mean fixation duration across categories (F_(2, 176)_ = 4.07, *p* = 0.02) that was due to longer fixations for GoalDir videos than ToM videos (Fig. 3: t_124_ = − 2.33, *p* = 0.021). However, like the group effects, this difference was opposite the hypothesized direction and disappeared when we included either peak motion (F_(2, 176)_ = 0.15, *p* = 0.861) or motion variability as a covariate (F_(2, 176)_ = 0.75, *p* = 0.473).

To examine viewing consistency within our group of monkeys we next looked at viewing patterns for each video within the categories. Individual monkeys did exhibit longer or shorter fixations on specific videos; however, these differences were idiosyncratic. Only one of the fifteen possible correlations between individual monkeys’ mean fixation durations (ms) was significant with another (Supplemental Fig. 1; Monkey CN vs. HI: r_10_ = 0.85, *p* = 0.0005).

**Figure 4:**
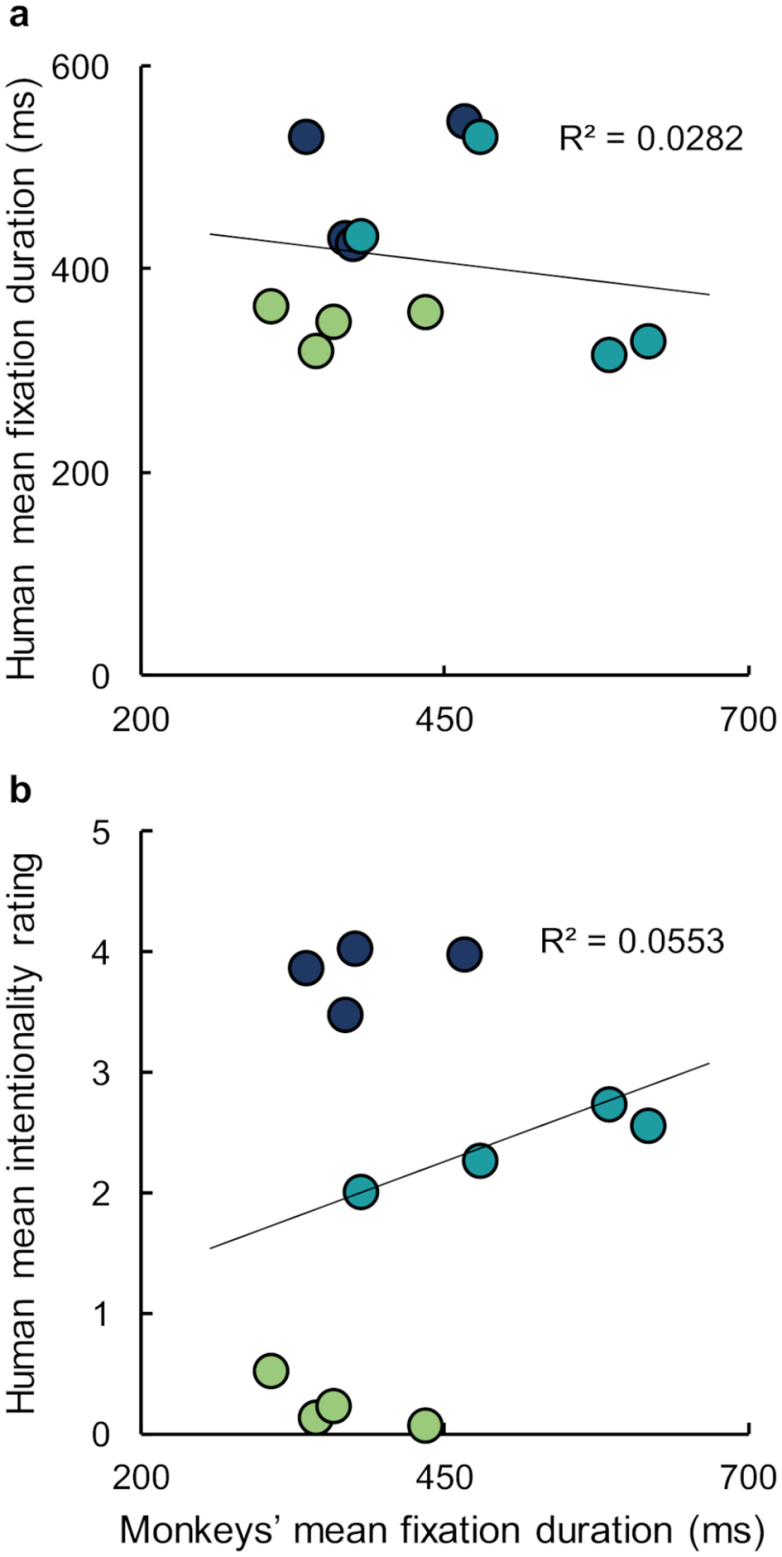
Monkeys’ mean fixation duration did not significantly correlate with humans’ fixation durations or intentionality ratings (data from Klein et al.^8^). (**a**) Monkeys’ mean fixation duration (ms) for each animation as a function of the humans’ mean fixation duration reported in Klein et al.^8^. (**b**) Monkeys’ mean fixation duration (ms) for each video as a function of humans’ intentionality ratings (0–5; 0 = nondeliberate action and 5 = deliberate action with the goal of affecting the other’s mental state). Each symbol represents data from a single animation. Colors represent animation categories, and match assignments used in Figs. 1, 2 and 3.

Comparing our data from monkeys to the published data from humans who viewed these same videos, monkeys’ mean fixation durations did not correlate with human’s mean fixation duration reported in Klein et al.^8^ (Fig. 4a: r_10_ = − 0.17, *p* = 0.60). Klein et al.^8^ also used verbal intentionality scores to rate video categories ranging from 0–5; 0 = nondeliberate action and 5 = deliberate action with the goal of affecting the other’s mental state. ToM elicited the highest intentionality rating. Importantly, humans' fixation durations were positively correlated with their intentionality ratings. Monkeys’ mean fixation durations did not correlate with human’s intentionality ratings (Fig. 4b: r_10_ = 0.24, *p* = 0.46).

The most conspicuous feature of our monkeys’ viewing patterns was their seeming disinterest in the Heider–Simmel animations. Monkeys spent only the minority of their time watching the videos (mean = 29.9%). The remainder was spent glancing around the testing room (personal observation), even though the testing room was familiar to them. This contrasts sharply with monkeys’ viewing patterns while watching more ethologically relevant videos, such as videos of conspecifics, food, or predators, for which monkeys in this same setup typically spend 60–80% of their time watching the videos (unpublished data). Nevertheless, we observed a similar pattern in total viewing time as we did with fixation duration, a significant main effect of category (F_(2,10)_ = 19.51, *p* < 0.001, partial η^2^ = 0.79) caused by modestly higher viewing of the GoalDir video category (mean(SEM): ToM = 25.3%(3.8%), GoalDir = 39.8%(4.1%), Rand = 24.7%(5.8%); ToM v GoalDir: t_5_ = − 6.54, *p* = 0.001; GoalDir v Rand: t_5_ = 4.85, *p* = 0.005). Viewing of ToM videos did not differ significantly from that of Random videos (t_5_ = − 0.21, *p* = 0.843). This difference between categories vanished when we included peak motion as a covariate (F_(2,10)_ = 3.06, *p* = 0.092) and was greatly reduced by including motion variability as a covariate (F_(2,10)_ = 4.88, *p* = 0.033).

## Discussion

To our knowledge, this is the first report of how nonhuman primates view the classic Heider–Simmel animations. Unlike humans, macaque monkeys did not show the pattern of fixating longest on ToM animations, next most on Goal Directed animations, and least on Random animations. Differences in fixation duration were inconsistent across monkeys. Critically, differences in fixation duration were not correlated with those previously observed in humans or with humans’ ratings of intentionality. Overall, these results provide no evidence that macaque monkeys spontaneously attribute mental states to abstract shapes based on their movement.

The unexpected group difference between ToM and Goal Directed videos we observed likely reflects a preference for low-level visual motion and is unlikely to reflect anything about ToM in monkeys. First, this finding was in the opposite direction we hypothesized based on the viewing duration in human subjects in Klein et al.^8^. Second, fixation durations for neither ToM nor Goal Directed videos differed from those of Random videos. Third, only one individual monkey’s viewing pattern reflected this group difference. Fourth, monkeys’ viewing patterns across animations were inconsistent with each other. Fifth, this difference went away both at the group level and the individual level when we included parameters of visual motion as covariates. Therefore, it seems most likely that the viewing difference between categories displayed by our monkeys was driven by the differences in low-level visual motion between stimuli that varied across categories.

Research using variants of free-viewing tasks suggests that at least some nonhuman primates attribute mental states to other animals but has found mixed evidence that mental state attribution spontaneously extends to abstract shapes based on motion, as it does in humans. Evidence for mental state attribution when interacting with other animals comes from findings that some primate species selectively attend to environmental constraints on others’ actions and react differently when those constraints render another’s action intentional versus accidental^16,27,28,30^ but see^31^. Evidence that mental state attribution spontaneously extends to animated shapes, as it does in humans, is scarcer. In great apes, chimpanzees and bonobos are reported to attribute mental states to animated shapes^17,40^. The study with chimpanzees suggested that the apes inferred intention to abstract shapes in a nonsocial context, and might, alternatively, be interpreted as apes having knowledge about object relations^17^. The study in bonobos used abstract shapes with eyes, giving them a distinctly social aspect^40^. Thus, these studies provide compelling but not yet uncontestable evidence that apes attribute mental states to animated abstract shapes. In monkeys, the evidence is more mixed and comes mostly from New World monkey species^36^. Squirrel monkeys might attribute goals to abstract shapes^37^. Findings from that study, however, were based on behavior learned with well-trained stimuli and thus are vulnerable to criticisms stemming from over-training. Few reports exist in Old World monkey species, such as rhesus macaques. Thus, while it is evident that some primate species attribute mental states to others, it is less clear the degree to which they spontaneously attribute mental states to inanimate objects, a characteristic of human ToM and apparent when viewing the Heider–Simmel animations.

Our findings are consistent with the idea that monkeys do not spontaneously attribute mental states to abstract animated shapes. Prior research has provided evidence that monkeys can represent others’ knowledge/ignorance but not others’ beliefs^18,26,32^, but see^34^. Much of these data come from false belief tasks that are competitive in design, which are limited by executive processing and inhibitory control capacities. There is conflicting evidence about the degree to which nonhuman primates, like humans, spontaneously attribute mental states to animated abstract shapes based on movement, with much of this evidence subject to the criticism that this ability is dependent on prior learning rather than spontaneous attribution^17,36,37^. Our evidence is most consistent with the prior work showing that monkeys attributed goals to a conspecific but not a moving black box^36^ and bolsters the conclusion that monkeys do not spontaneously attribute mental states to inanimate objects based on movement.

This study has three major limitations. First, it reports data from a relatively low number of subjects compared to human studies. However, the number of subjects is comparable to the other studies of how monkeys view animated shapes^37,38^. Further, if we assume the large effect size reported for humans’ difference in fixation durations^8^, an a priori power analysis suggests that we would need only three subjects to achieve a power of 0.9 with our repeated-measures design, suggesting that we would have detected an effect if it were present. Second, this study was designed to evaluate whether an overall effect of video category on fixation duration might exist in monkeys, as it does in humans, and so cannot robustly assess the moderating effects of other factors such as viewing order, subject dominance, or subject sex. Had we observed a human-like viewing pattern, we would have explored such factors in follow-up experiments, but this was not necessary. Third, and most important, is that the interactions in the Heider–Simmel animations are modeled after human social behavior and may not be ethologically relevant to nonhuman primates. Shape movements suggesting “mocking” or “seduction” may not elicit enhanced social processing in monkeys because human-like mockery and seduction are not part of monkeys’ social repertoire. Goal Directed animations such as “chasing” might be more salient to monkeys’ behavioral repertoire, possibly explaining why we observed the increased viewing time for that category (though the explanation based on stimulus movement remains most parsimonious given the current evidence). Future studies of how rhesus monkeys view animations that resemble macaque social behavior might provide a better test of whether monkeys ascribe mental states to animated shapes. Nonetheless, assessing monkeys’ viewing patterns on these classic stimuli, especially using the same metric as has been used in humans and that correlates with humans’ verbal reports, remains a valuable piece of evidence to inform the broader question of nonhuman ToM.

It is likely that ToM benefits substantially from language and data from nonhuman species can inform our understanding of this benefit. Fully-developed language is clearly not necessary for all aspects of ToM; pre-verbal infants can derive relational causality and goal directed behavior from animated shapes and inanimate objects with relatively little experience^3–5,41,42^. However, humans are a fundamentally verbal species, making it difficult to disentangle language from any of our cognitive abilities. We do not claim that evidence from monkeys is the ultimate test of the contribution of language. Rather that understanding the overlap between human and nonhuman ToM is one informative piece of evidence about language, and testing monkeys using the same stimuli and metric as used in humans informs the degree of that overlap.

Yet another possibility is that the Heider–Simmel task requires a level of abstract representation that is specific to humans. For example, attributing mental states to animated objects may require a form of analogical reasoning (big triangle:little triangle::mother:child). Indeed, others have suggested that the ability to “interpret the world in a symbolic-relational fashion” is what most separates the cognition of humans from the cognition of other animals^43^. In our study, macaque’s seeming failure to attribute mental states to abstract shapes may be less about their inability to attribute mental states and more about their inability to relate arbitrary, moving symbols in the physical domain to the social domain.

A related possibility is that the Heider–Simmel animations represent a very narrow test of a niche form of ToM. Instead of testing only our ability to recognize intentions, they may test our ability to tell stories about intention or create verbal representations of abstract animations. For example, for a description to be accurate in Abell et al.^7^ for the ToM videos participants had to assign character roles and create hypothetical scenarios (e.g., the child is reluctant to go out and the mother attempts to get the child out). In the “coaxing” example, children had to denote the larger triangle as having more authority (the mother) and assign a role to the smaller triangle that related to the other figure. They had to create a narrative for shapes that was likely informed by their own experiences. By contrast, getting an accurate score for the Goal Directed videos did not require a high level of creativity or sense of personal relationships as children just had to deduce the relationship of movement between the two objects^7^, which nonhuman primates have demonstrated the ability to do in different contexts^16,17,30,36,37^. Thus, this may indicate that monkeys lack the verbally dependent storytelling aspect of ToM but not necessarily other aspects that do not require a high cognitive load or creative component.

In summary, we found that macaque monkeys did not show evidence of attributing mental states to animated shapes when viewing the classic Heider–Simmel videos. This evidence does not rule out the possibility that monkeys may be able to demonstrate these skills on tasks not requiring narrative capacities or tasks that involve interactions with conspecifics or animations depicting more relevant social behavior. However, this evidence does suggest that humans’ tendency to anthropomorphize animated shapes is specific to human, and perhaps ape, cognition.

## Methods

### Subjects and apparatus

Six adult male rhesus monkeys (*Macaca mulatta)*, with a mean age of 8.6 years at the start of the study, served as subjects. The monkeys were housed individually, had visual and auditory contact with conspecifics, and were kept on a 12-h light–dark cycle. Under veterinary supervision, we controlled monkeys’ water intake to maintain both motivation and health.

The test apparatus comprised a booth, a 15″ display monitor, generic audio speakers, an eye-tracking camera (Arrington Research; PC60 camera), an infrared light source, and a juice-delivery system^44^. During testing, monkeys sat comfortably in a behavioral testing chair with their heads fixed. They faced a display monitor and eye-tracking camera; the distance between the monkey's head and the monitor was approximately 57 cm (Fig. 1a). Animations were displayed through Presentation (Neurobehavioral Systems). This study was carried out in accordance with the Guide for the Care and Use of Laboratory Animals and the US Animal Welfare Act. The protocol was approved by the National Institute of Mental Health Animal Care and Use Committee.

### Stimuli

We used the same animation categories as those used by Abell et al.^7^ and Klein et al.^8^: Theory of Mind (ToM), Goal Directed (GoalDir), and Random (Rand). Each category contained four videos (Fig. 1b). In ToM animations, the objects interacted in a complex way that indicated one object acknowledged and reacted to the other object’s mental state (e.g., coaxing, surprising, seducing, and mocking). In GoalDir animations, the objects moved in a purposeful interactive way to elicit a straightforward outcome (e.g., chasing, fighting, leading, and dancing). Lastly, in Rand animations, the two objects did not interact and moved purposelessly. Note that Random animations 1–4 correspond to the labels “tennis”, “billiards”, “drifting”, and “star” used in previous studies.

### Procedure

Each monkey first completed a nine-point calibration to relate eye-tracker output to gaze points on the screen. Monkeys received 0.1 ml of 50% juice:water mix each time they looked at a calibration point. The monkeys then viewed the Heider–Simmel animations. The experiment consisted of twelve trials in which the twelve animations were presented in a random order with the constraint that each block of the three trials contained one animation of each category. Each animation lasted 18 s and was followed by a 30–40 s ITI (Fig. 1c). Each animation was presented once, and monkeys had no prior exposure to them. Monkeys freely viewed animations and did not receive additional juice until after the session. The single test session lasted approximately 15 min.

### Data analysis

We classified eye movements as fixations if they did not exceed a velocity of 100 degrees/second and if they maintained quality tracking of both pupil and infrared glint in the Arrington software. As with humans^8^, mean duration of individual fixations was our central measure. Because the study directly comparable to ours used parametric analyses, we did the same: analyzing differences between categories with repeated measures ANOVA followed by paired t-tests for group performance and single factor ANOVA followed by independent t-tests for each individual subject’s performance. However, because fixation duration measures are usually non-normally distributed—which was the case in this study and probably the case in the previous study of human subjects as well—we also ran nonparametric equivalents: Friedman’s tests followed by Wilcoxon’s signed-rank tests for group analyses and Kruskal–Wallis tests followed by Mann–Whitney U tests for individual analyses. Both parametric and nonparametric analyses returned the same results, thus we report the parametric tests to facilitate comparison with the study of humans^45^. Because these stimuli were not matched for motion, and visual inspection suggested that the Rand videos had smaller, more uniform motion than the ToM and GoalDir videos, we assessed peak motion and motion variability in the videos using published methods^39^ and used those motion variables as covariates where appropriate. Motion variability was the standard deviation of the mean pixel motion of each frame from the previous frame and peak motion was the maximum frame-to-frame change. All tests were two-tailed with *α* = 0.05 unless noted. We examined the consistency of viewing between individual monkeys via Pearson’s correlations. Lastly, we examined the relation between the viewing patterns of our monkeys and the viewing patterns and intentionality ratings reported for normal humans^8^ via Pearson’s and Spearman’s correlations, respectively.

## Supplementary information

Supplementary Information

Supplementary Videos
