# Supplementary material for: No evidence that monkeys attribute mental states to animated shapes in the Heider–Simmel videos
Source: Sci Rep. 2021 Feb 4;11:3050. doi: 10.1038/s41598-021-82702-6 (PMC7862678; doi:10.1038/s41598-021-82702-6)
Supplement: Supplementary file 1 — Supplementary Information [file 41598_2021_82702_MOESM1_ESM.pdf]

Supplemental material for

No evidence that monkeys attribute mental states to animated shapes in the Heider-Simmel videos

Jamie L. Schafroth, Benjamin M. Basile, Alex Martin, and Elisabeth A. Murray

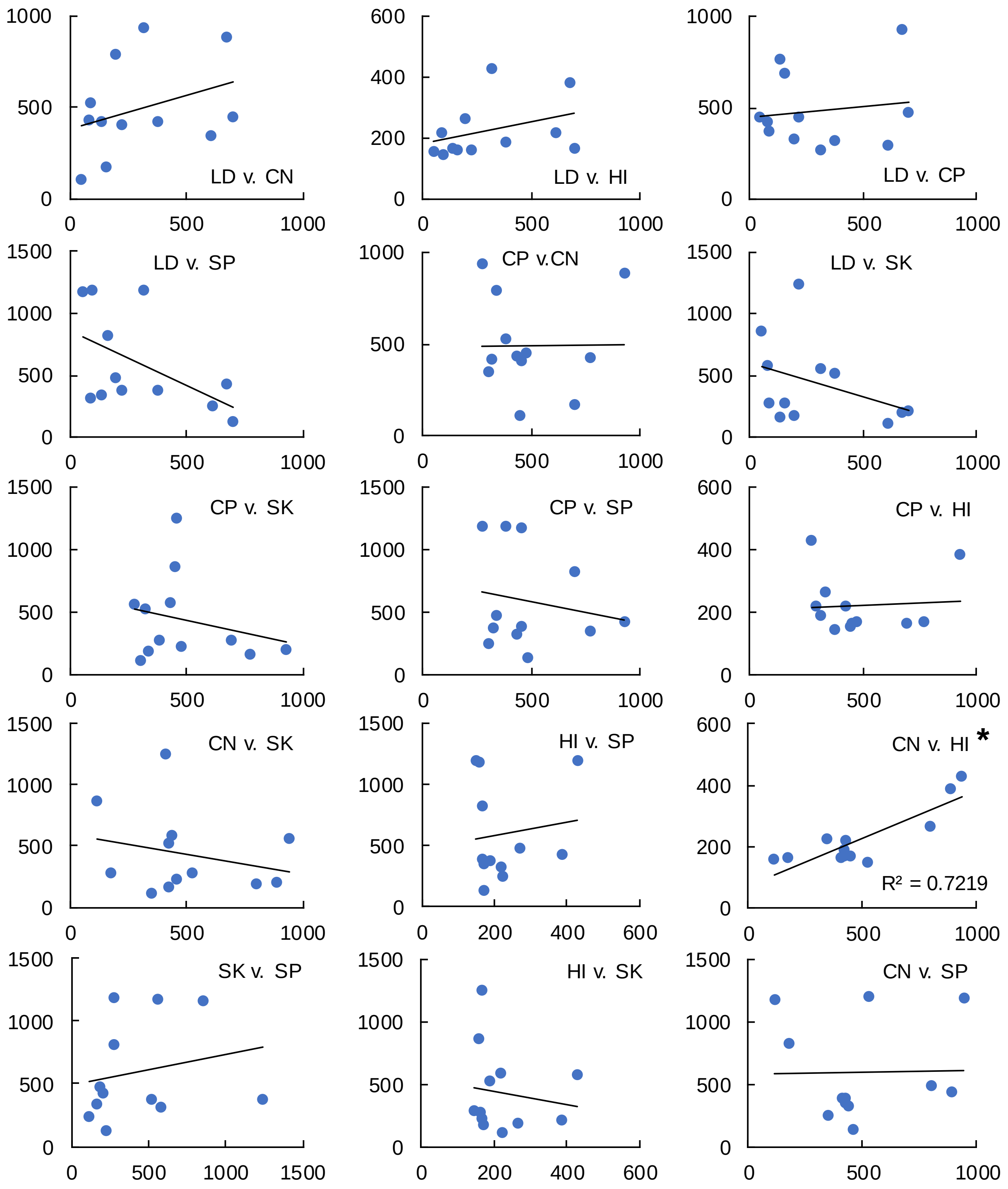

Supplemental Figure 1. Differences in fixation duration were not consistent across monkeys. Each monkey's viewing pattern as a function of each other monkey. The first monkey of each pair is on the x axis and the second on the y axis. Each marker represents a video. * = $p < .05$.

Supplemental Videos. The Heider-Simmel animations used in this study. Filenames correspond to Figure 1, with the exception that random animations 1-4 correspond to the labels “tennis”, “billiards”, “drifting”, and “star”. Videos here are lower quality than were used in the actual study.
